# Multi-parametric radiomics of conventional T1 weighted and susceptibility-weighted imaging for differential diagnosis of idiopathic Parkinson’s disease and multiple system atrophy

**DOI:** 10.1186/s12880-023-01169-1

**Published:** 2023-12-08

**Authors:** Shuting Bu, Huize Pang, Xiaolu Li, Mengwan Zhao, Juzhou Wang, Yu Liu, Hongmei Yu

**Affiliations:** 1 Department of Radiology, the First Hospital of China Medical University, Shenyang, 110001 China; 2 Department of Neurology, the First Hospital of China Medical University, 155 Nanjing North Street, Shenyang, Liaoning 110001 PR China

**Keywords:** Idiopathic Parkinson’s disease, Multiple system atrophy, Radiomics, MRI, Light GBM

## Abstract

**Objectives:**

This study aims to investigate the potential of radiomics with multiple parameters from conventional T1 weighted imaging (T1WI) and susceptibility weighted imaging (SWI) in distinguishing between idiopathic Parkinson’s disease (PD) and multiple system atrophy (MSA).

**Methods:**

A total of 201 participants, including 57 patients with PD, 74 with MSA, and 70 healthy control (HCs) individuals, underwent T1WI and SWI scans. From the 12 subcortical nuclei (e.g. red nucleus, substantia nigra, subthalamic nucleus, putamen, globus pallidus, and caudate nucleus), 2640 radiomic features were extracted from both T1WI and SWI scans. Three classification models - logistic regression (LR), support vector machine (SVM), and light gradient boosting machine (LGBM) - were used to distinguish between MSA and PD, as well as among MSA, PD, and HC. These classifications were based on features extracted from T1WI, SWI, and a combination of T1WI and SWI. Five-fold cross-validation was used to evaluate the performance of the models with metrics such as sensitivity, specificity, accuracy, and area under the receiver operating curve (AUC). During each fold, the ANOVA and least absolute shrinkage and selection operator (LASSO) methods were used to identify the most relevant subset of features for the model training process.

**Results:**

The LGBM model trained by the features combination of T1WI and SWI exhibited the most outstanding differential performance in both the three-class classification task of MSA vs. PD vs. HC and the binary classification task of MSA vs. PD, with an accuracy of 0.814 and 0.854, and an AUC of 0.904 and 0.881, respectively. The texture-based differences (GLCM) of the SN and the shape-based differences of the GP were highly effective in discriminating between the three classes and two classes, respectively.

**Conclusions:**

Radiomic features combining T1WI and SWI can achieve a satisfactory differential diagnosis for PD, MSA, and HC groups, as well as for PD and MSA groups, thus providing a useful tool for clinical decision-making based on routine MRI sequences.

**Supplementary Information:**

The online version contains supplementary material available at 10.1186/s12880-023-01169-1.

## Introduction

Idiopathic Parkinson’s disease (PD) and multiple system atrophy (MSA) are two common neurodegenerative conditions, which share overlapping Parkinson’s motor symptoms, particularly in the early stages [1]. Clinicians often face a major challenge in differentiating between PD and MSA patients due to the reliance on subjective factors such as symptoms, physical examination, and the expertise of the neurologist for clinical diagnosis. These subjective assessments may be susceptible to personal bias, leading to diagnostic uncertainty. In comparison to PD, MSA is characterized by a more rapid progression and poorer prognosis. Therefore, the development of a practical and sensitive diagnostic tool is crucial for accurate differentiation between these two conditions [2]. Different signs on conventional Magnetic Resonance Imaging (MRI) like the “swallow tail” sign, “putaminal hypointensity”, and “hot cross” sign, which are influenced by the magnetic field strength and the sequence type, provide additional support for the diagnosis between PD and atypical Parkinson syndrome (APS) [3–5]. However, it is not easy for radiologists to make a diagnosis when the change is subtle. To this end, it is essential to create objective and convenient biomarkers for clinical diagnosis.

The utilization of advanced MRI, with its various modalities having various tissue-specific sensitivities, has been demonstrated to be advantageous in detecting both PD and APS. For example, resting-state functional magnetic resonance imaging (rs-fMRI) and diffusion magnetic resonance imaging (dMRI) have shown great potential in detecting subtle functional and structural alterations in patients with PD and MSA [6, 7]. Although the specific findings of advanced MRI appear promising, their clinical application is currently limited due to the extended scanning period and complex processing pipelines involved. T1 weighted imaging (T1WI) and T2 weighted imaging (T2WI), as the most conventional sequence, reflect anatomical and pathological information of the disease. In clinical practice, T1WI has been extensively utilized to detect morphological changes in the brain of patients with PD and APS using various processing methods. Previous studies have demonstrated that PD patients exhibit a decrease in gray matter volume in specific brain regions compared to HCs, including the basal ganglia (putamen and caudate nucleus), theory of mind (temporal lobe, amygdala, superior frontal gyrus and anterior cingulate gyrus), vocal (temporal lobe, rolandic operculum, insula and putamen) and visual network (middle occipital gyrus and fusiform gyrus), temporal, parietal, and postcentral regions [8–11]. While some researchers reported that cognitively intact PD patients do not show significant gray matter alterations [12]. In addition, other studies revealed a more widespread pattern of brain atrophy in patients with MSA, which includes the striatum, prefrontal cortex, cerebellum, pons, thalamus (Tha), putamen (PUT), and midbrain [13, 14]. However, visible brain atrophy may be a symptom of advanced disease, making it difficult to diagnose in the early stage of the disease. Susceptibility-weighted imaging (SWI), which is derived from a T2*-weighted gradient-echo sequence, is a valuable tool for detecting iron deposition. It has been extensively utilized in clinical diagnosis due to the fact that iron accumulation in the basal ganglia is considered a significant factor in the pathogenesis of PD. Different iron deposition patterns have been suggested for PD and APS, which primarily involve the substantia nigra (SN), red nucleus (RN), Tha, PUT, and caudate nucleus (CN) [15, 16]. Nevertheless, it is important to note that there have been inconsistencies and discrepancies found in the literature regarding the role of iron deposition and its pattern. Furthermore, the visual evaluation of iron deposition on SWI may be challenging in the initial stage and can also be observed in the brains of healthy elderly individuals, leading to an increased rate of false positives in clinical diagnoses. Therefore, it becomes crucial to accurately detect even subtle and early brain alterations in PD and MSA patients to ensure precise diagnoses and appropriate management strategies.

Radiomics is an emerging technique that enables the extraction of quantitative data from medical images, including information that may not be visually apparent. This technique holds great potential for early diagnosis in various fields, including neurodegenerative diseases such as Alzheimer’s Disease (AD) and PD [17, 18]. Recently, Tupe et al. applied radiomics analysis based on several gray matter regions in the cerebrum and cerebellum on T1WI, and presented an accuracy of 92% in distinguishing PD from APS [19]. Aside from gray matter, Shu et al. focused on the white matter derived from T1WI. Radiomics features were derived specifically from the white matter regions and had a desirable performance (AUC = 0.836) for progression prediction in PD patients [20]. Meanwhile, other investigators attempted to make radiomics analyses based on the SWI sequence. For example, Pang et al. extracted radiomics features on SWI, and achieved an AUC of 0.862 in the differential diagnosis between PD and parkinsonian variants of MSA (MSA-P ) [18]. To date, no research has been conducted to examine the impact of radiomics analysis based on the combination of conventional sequence (T1WI and SWI) in differentiating PD from MSA. Nevertheless, according to prior studies, the combination of multiple sequences to evaluate the underlying pathophysiology may lead to a more precise understanding of the disease and an accurate prediction [21, 22]. Both T1WI and SWI are widely employed in clinical practice. Consequently, the application of radiomics analysis, which integrates these two imaging modalities, holds the potential to provide supplementary advantages for differential diagnosis. Additionally, previous studies focused solely on a single machine learning method, lacking of the comparison of diagnostic performance between different models.

Specifically, the purpose of this study was to build a radiomic model of best performance based on features derived from basal ganglia regions, using commonly applied sequences in clinical settings, to distinguish between PD and MSA.

## Material and methods

### Subjects and clinical assessment

This retrospective study was approved by the Ethics Committee of the First hospital of China Medical University and informed consents were obtained from each subject, and the Declaration of Helsinki were followed at all stages of study (decision date and number: 04.07.2020, No.AF-SOP-07-1.1-01). Overall, 74 patients with MSA, 57 patients with PD were retrospectively recruited between June 2017 and December 2020, and 70 HCs matched for age, gender were enrolled from the local community at the same time. The inclusion criteria were as follows: (1) the patients visited in our hospital met the diagnostic criteria for PD and MSA, which follows the UK PD Society Brain Bank [23] and the “probable MSA” via second-consensus clinical criteria [24], respectively. (2) the patients underwent both T1WI and SWI. (3) disease duration was within 5 years, with average over 2 years of follow-up. The exclusion criteria were as follows:(1) medical history including a past of substance abuse, endocrine disease, or the thyroid disorders. (2) neurological illnesses or pathological findings on conventional MRI. (3) other diseases that may cause abnormal iron deposition. (4) image artifacts. (5) missing or incomplete clinical or imaging data. A series of clinical evaluations, which included movement disorders and cognitive conditions, were measured by part III of the Unified Parkinson’s Disease Rating Scale (UPDRSIII) and Montreal Cognitive Assessment (MoCA), respectively. To address potential ethical and privacy concerns, all the data of recruited subjects have been anonymized.

### MRI protocol

All patients underwent conventional 3.0 T MRI examination (Magnetom Verio, Siemens, Erlangen, Germany), which includes T1WI and SWI sequence. All participants were scanned parallel to the anterior commissure-posterior commissure (AC-PC) plane. High-resolution three-dimensional sagittal magnetization-prepared rapid acquisition gradient echo (MPRAGE) T1-weighted sequence was acquired with the following parameters: repetition time (TR) = 5000 ms, echo time (TE) = 2960 ms, flip angle (FA) = 12°, field of view (FOV) = 256 × 256mm^2^, matrix size = 256 × 256, slice thickness = 1 mm, no slice gap, voxel size = 1.0 × 1.0 × 1.0mm^3^, and slice number = 176. The SWI was obtained using parameters as follows: TR = 27 ms, TE = 20 ms, slice number = 64, slice thickness = 0.8 mm, FA = 15°, FOV = 230 × 172.5 mm^2^, matrix size = 0.9 × 0.9 × 0.8mm^3^.

### ROI segmentation and image processing

According to previous studies on iron deposition patterns in PD and APS, regions of interest (ROIs) have been established, which includes RN, SN, CN, PUT, globus pallidus (GP), and subthalamic nucleus (STN) [15, 18]. Considering basal ganglion is well delineated on SWI compared with T1WI, volumes of interest (VOI) were conducted on sequential layers on SWI by an experienced radiologist with more than 5 years of experience in neurology diagnosis using ITK-SNAP (version 3.6.0, www.itksnap.org). To examine the reliability of the SWI data, the identical radiologist performed VOI segmentation using the same methodology for intra-observer agreement assessment after a month. Simultaneously, another experienced radiologist independently performed VOI segmentation using the same methods to evaluate inter-observer reliability. Intra- and inter-observer correlation coefficients (ICCs) were employed to quantify observer agreement in the extraction of radiomics features from the VOIs [25]. Then ROIs on SWI were linearly registered to individual T1WI and the quality of the registrations was manually assessed. Co-registration procedure was performed using advanced normalization tools (ANTs).

### Feature extraction

For each ROI delineating 6 types of nuclei in two hemispheres (a totally of 12 nuclei, “1” denotes left side, “2” denotes right side) on T1WI or SWI, a total of 110 features, consisting of first-order image intensity statistics, shape and texture features (gray level cooccurrence matrix (GLCM), gray level run length matrix (GLRLM), gray level size zone matrix (GLSZM), gray level dependence matrix (GLDM), and neighboring gray tone difference matrix (NGTDM)), were automatically extracted using the open-source Python package of Pyradiomics [26]. These features were obtained through 14 image filters accomplished by SimpleITK filters (ie, original image, Laplacian sharpening, discrete Gaussian, shot noise). Following the extraction of radiomic features, each feature was normalized using the min-max normalization method, which rescaled the values to a range of 0 to 1.

### Feature selection and modeling

Considering the size of sample and the desired trade-off between computational cost and accuracy, a five-fold cross-validation strategy was employed in the experiment. Briefly, in each round of cross-validation, all the data was equally split into five subsets, often referred to as “folds”. Four subsets were used as the training set and the rest one was used as the testing set alternatively. Moreover, 70% of training data points were utilized to train the model, and the remaining 30% were utilized to validate and select the best model [27, 28]. In each fold, before building the model, the most relevant feature subset was first selected from abundant features in the training data to reduce the feature dimension and avoid overfitting. Firstly, analysis of variance (ANOVA) was applied to exclude the feature, whose standard value is smaller than 0.01. The standard indicates the degree of dispersion. The smaller the standard value is, the less distinguishable the feature is. Secondly, spearman correlation analysis was performed to reduce the collinearity of features. When the correlation coefficient between two features was r > 0.9, one feature was randomly retained. Lastly, LASSO was performed to reduce the unimportant features and select the most representative features in the training dataset with non-zero coefficient values. The best parameter was determined by the grid search algorithm.

**Fig. 1:**
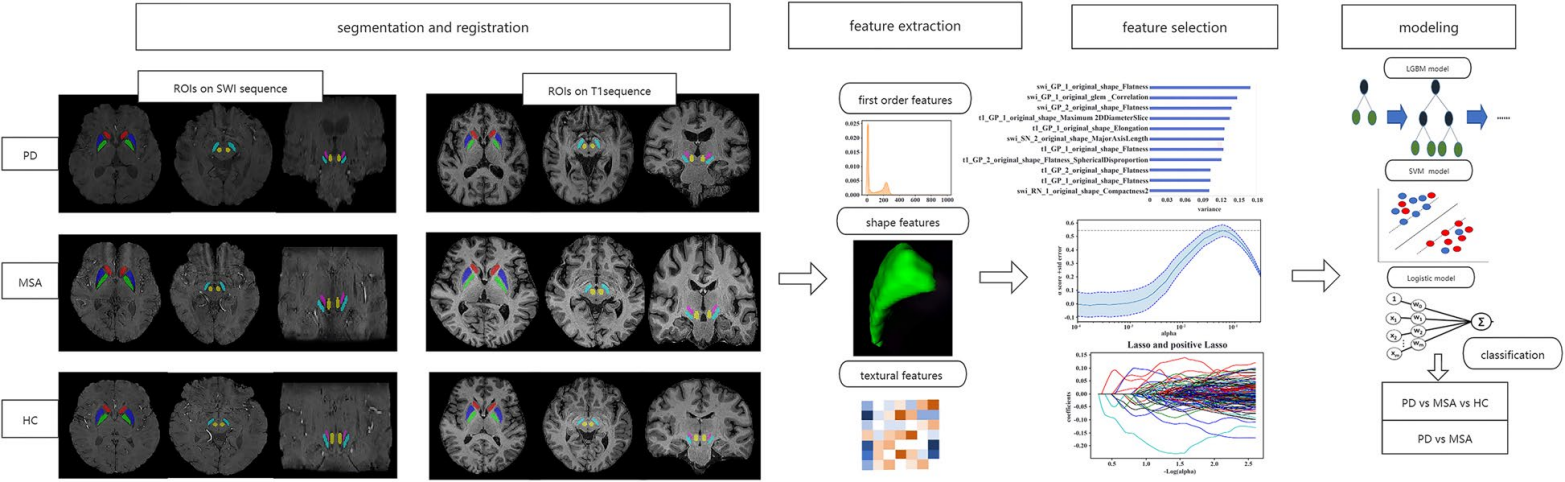
The workflow of this study (1) Segmentation and registration: Regions of caudate, putamen, globus pallidus, substantia nucleus, red nucleus, and subthalamic nucleus were manually segmented on SWI sequence, and co-registered to T1 sequence. (2) Feature extraction: Three kinds of features were extracted from SWI and T1 sequence. (3) Feature selection: ANOVA and lasso were applied for feature selection procedure. (4) Modeling: Three different classification models (logistic regression (LR), support vector machine (SVM), and light gradient boosting machine (LGBM)) were built to distinguish different groups: including MSA vs. PD, and MSA vs. PD vs. HC

After feature selection procedure, the retained features were used as the input of classifiers. With the selected features, three different classifiers were constructed and compared using each of the five folds, which include logistic regression (LR), support vector machine (SVM) with radial basis function kernel (RBF), and light gradient boosting method (LGBM). After feature selection, the features of single-parametric sequence named T1WI, the features of single-parametric sequence named SWI, and the features combination of multi-parametric sequences of T1WI and SWI, were adopted to build the classification model, respectively. The classification tasks include the three-category: HC vs PD vs MSA, and binary category: PD vs MSA. Besides, Shapley additive explanations (SHAP) values of each feature were computed to understand the most valuable features in prediction. SHAP, as a game-theoretic approach, interprets the output of machine learning model by calculating features’ contributions. The feature processing, selection, and classifier construction were performed on the Anaconda3 platform (www.anaconda.com) with the “scikit-learn” package (scikit-learn.org) using Python version 3.7.4. Figure 1 presents the workflow of this study.

### Statistical analysis

Numerical clinical characteristics among HCs, PD, and MSA were evaluated using ANOVA. The assessment of discrete data was performed using the chi-square test. The Kruskal-Wallis test, accompanied by the Dunn multiple comparisons test, was used for data exceeded homogeneity test for variance. The Mann-Whitney test and independent two-sample t-test were conducted to assess the differences between PD and MSA patients for various phenotype data, as deemed appropriate. All statistical analyses were two-sides, and *P* < 0.05 was considered statistically significant. The statistical analyses were performed using SPSS23.0. The diagnostic performance of classifiers was evaluated using the area under the receiver operating characteristic (ROC) curve (AUC), as well as measures of accuracy, sensitivity, and specificity. Then Delong test was used to exam diagnostic performance in different classifiers.

## Results

### Clinical characteristics

There were no significant differences in age and gender among three groups. The MSA and PD groups had significantly lower MoCA scores compared with HCs (*p* < 0.001). No significant differences were observed in motor UPDRSIII scores and cognitive MoCA scores between the patient groups, and PD showed statistically significant in age compared with MSA (*p* = 0.04). While the MSA patients had significantly shorter disease duration in comparison with PD groups (*p* < 0.001) (Table 1).

**Table 1:** Demographic and clinical characteristics of participants

Characteristics	Patients (*n* = 131)		HC (*n* = 70)	U/χ^2^	*P* value
	PD (*n* = 57)	MSA (*n* = 74)			
Age (years)	63.47 ± 7.85^a^	65.96 ± 6.51	64.97 ± 6.66	2.06	0.13
Gender (M/F)	30/27	35/39	34/36	0.387	0.82
Disease duration	2.88 ± 1.56	2.27 ± 0.92	N	21.474	< 0.001^***^
UPDRSIII score	42.42 ± 6.14	43.19 ± 8.59	N	−0.60	0.55
MoCA score	22.93 ± 2.17^b^	22.16 ± 2.20^c^	26.99 ± 1.52	119.93	< 0.001^***^

*Disease duration* Duration from onset of PD symptoms to scan (Years)

***denotes *p* value less than 0.001

^a^denotes *p* value less than 0.05 between PD and MSA

^b^denotes p value less than 0.05 between PD and HC

^c^denotes p value less than 0.05 between MSA and HC

### Feature selection and model performance

After performing ANOVA, all *P* values of radiomic features among three groups were less than 0.05, thus none of the features were removed. Next, LASSO was applied using five-fold cross-validation, and 362 features were left in total five cross-validation folds. Generally, the performance of models based on the combination of T1WI and SWI sequences outperformed single sequence (Fig. 3, Table 3).

**Fig. 2:**
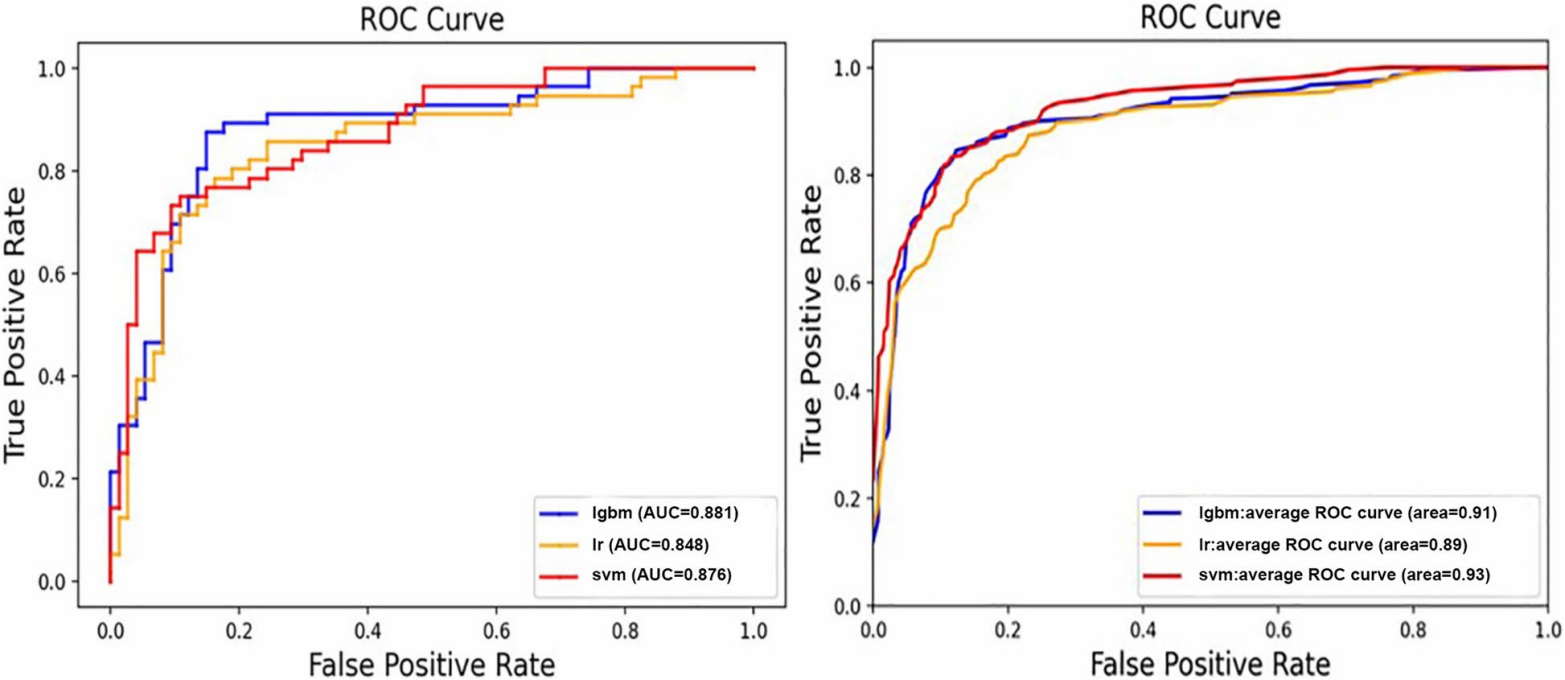
Receiver operator curves (ROC) of different models based on radiomic features extracted from the combined T1WI and SWI sequence in binary and three-way classification. Left: differential diagnosis of PD and MSA. Right: differential diagnosis among PD, MSA, and HC. lgbm, light gradient boosting machine; lr, logistic regression; svm, support vector machine

As for the different models, the performances of the three classifiers were not all the same. The classifiers show an accuracy of 0.724 to 0.814, with an AUC of 0.869 to 0.926 in three-classification task (Table 2, Fig. 2).

**Table 2:** The performance of different models using T1 and SWI sequence in three-classification tasks (PD vs MSA vs HC)

Sequence	Models	Average			
		Sen	Spec	ACC	AUC
SWI	LR	0.762	0.882	0.764	0.869
	SVM	0.787	0.896	0.789	0.914
	LGBM	0.792	0.897	0.794	0.917
T1	LR	0.736	0.872	0.744	0.839
	SVM	0.712	0.861	0.724	0.882
	LGBM	0.723	0.864	0.729	0.887
SWI + T1	LR	0.745	0.875	0.749	0.885
	SVM	0.798	0.903	0.804	0.914
	LGBM	0.812	0.907	0.814	0.905

*Sen* sensitive, *Spec* specificity, *ACC* accuracy, *AUC* area under the curve, *SWI* susceptibility weighted imaging, *T1* T1 weighted imaging, *LR* logistic regression, *SVM* support vector machine, *LGBM* light gradient boosting machine

**Fig. 3:**
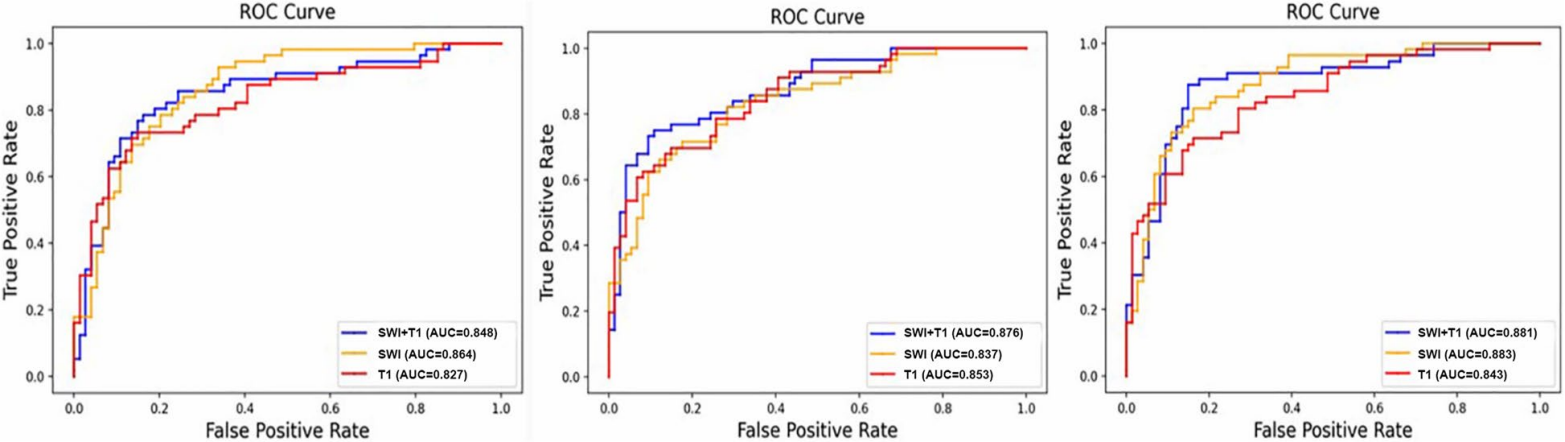
Receiver operator curves (ROC) of different models based on radiomic features extracted from T1 weighted imaging (T1WI) and susceptibility weighted imaging (SWI) sequence in the differential diagnosis of idiopathic Parkinson’s disease (PD) and multiple system atrophy (MSA) Left: logistic regression (LR); Middle: support vector machine (SVM); Right: light gradient boosting machine (LGBM)

**Fig. 4:**
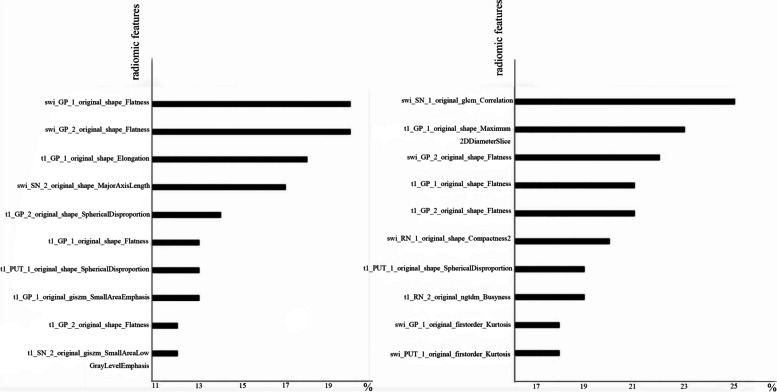
The first ten importance-ordered radiomic features extracted from combined sequences in each fold in the LGBM model of binary and three classification tasks. Left: PD vs. MSA; Right: PD vs. MSA vs. HC.  swi, susceptibility weighted imaging; t1, T1 weighted imaging; GP, globus pallidus; SN, substantia nigra; PUT, putamen; RN, red nucleus; 1, left; 2, right

Meanwhile, the models manifested an accuracy of 0.738 to 0.854, with an AUC of 0.827 to 0.883 in the binary classification task (Table 3, Fig. 3). In general, the LGBM model exhibited superior performance compared to the other classifier in both three classification and binary classification tasks, although there were no statistical differences between any two models when assessed using the Delong test. Besides, the first 10 importance-ordered features extracted from combined sequences (T1 and SWI) in each fold were shown in Fig. 4. The GLCM_correlation of SN and original_shape_flatness of GP served as the most stable and significant features in PD vs MSA vs HC and PD vs MSA, respectively.

**Table 3:** The performance of different models using T1 and SWI sequence in binary classification tasks (PD vs MSA)

Sequence	Models	Average			
		Sen	Spec	ACC	AUC
SWI	LR	0.750	0.811	0.785	0.864
	SVM	0.714	0.797	0.769	0.837
	LGBM	0.768	0.851	0.815	0.883
T1	LR	0.732	0.743	0.738	0.827
	SVM	0.696	0.824	0.769	0.853
	LGBM	0.714	0.811	0.769	0.844
SWI + T1	LR	0.821	0.757	0.785	0.848
	SVM	0.768	0.824	0.800	0.876
	LGBM	0.857	0.851	0.854	0.881

*Sen* sensitive, *Spec* specificity, *ACC* accuracy, *AUC* area under the curve, *SWI* susceptibility weighted imaging, *T1* T1 weighted imaging, *LR* logistic regression, *SVM* support vector machine, *LGBM* light gradient boosting machine

## Discussion

Conventional MR imaging techniques, such as T1WI and SWI, serves as the primary screening and diagnostic tools for clinical decision-making in the cases of PD and MSA [1, 29]. However, due to the similarities in imaging between patients with these two conditions, even experienced radiologists may have difficulties in distinguishing between them based solely on these images [30]. Besides, early detection and precise diagnosis play a vital role in enhancing the quality of life for individuals affected by these neurodegenerative diseases [2]. Recent studies also demonstrated that machine learning models based on radiomic features, deep features and clinical features have achieved a vital predictive performance [31, 32]. As such, this study has developed a radiomic analysis based on radiomic features extracted from conventional MR images, with the aim of precisely identifying patients with PD and MSA.

Previous studies applied radiomic analysis on either T1WI or SWI. For instance, Peng et al. conducted a study exploring the effectiveness of a SVM classifier employing multilevel ROI features based on T1WI, achieving an accuracy of 85.78% in the diagnosis of PD [33]. In another study, Vitali et al. proposed that T1-weighted volumetry of the SN on magnetization transfer-prepared MRI could serve as a helpful tool for staging PD [34]. Additionally, Shu et al. extracted radiomic features from white matter volumes on T1WI to predict the progression of PD [20]. Except for T1WI, researchers have also explored the use of iron-sensitive sequences in building radiomic models. For instance, Xiao et al. employed convolutional neural network (CNN) features and radiomic features of the SN on quantitative susceptibility mapping (QSM) to identify PD and achieved a remarkable diagnostic accuracy [35]. Aside from single sequence, there is a growing interest in utilizing multimodal MRI sequences for diagnosis. For example, Peran et al. applied multimodal MRI indices extracted from T2*, T1 weighted, and diffusion tensor imaging (DTI), to differentiate PD from MSA, achieving a high diagnostic accuracy [36]. Similarly, Chougar et al. developed a differential diagnosis model based on volumetry and diffusion metrics obtained from T1WI and DTI, which demonstrated a high accuracy in distinguishing PD from APD [37]. Aside from DTI, researchers have applied radiomics models based on functional MRI (fMRI) and discovered that cerebellar connectivity shows potential in differentiating PD from MSA [38]. However, DTI and fMRI are not commonly used in clinical practice due to its long scan time and complex process methods. The utility of T1 and SWI is valuable in diagnosing those patients with motor symptoms and improving the acceptance of MRI examination for its shorter scan time. Our study is the first to build radiomic models based on radiomic features extracted from the most widely-applied T1WI sequence as well as SWI sequence in clinical.

It is not surprising that models based on the combination of both T1 and SWI sequences outperformed those of the single sequence, although no statistical significance was observed in our study. Studies have suggested that the combination of multiple sequences, which evaluate the underlying pathophysiology more comprehensively, can result in a better understanding of the disease and a more accurate prediction [21]. In our study, we employed three models to differentiate PD from MSA and HCs, namely LR, SVM, and LGBM. Although the performance of these models may be similar, LGBM appears to be the most reliable and outperformed the other two models. LGBM is a novel decision tree algorithm based on a gradient boosting decision tree, which consists of multiple decision trees. Each tree divides the data into two groups based on features. LGBM has been demonstrated to be a powerful and effective model due to its limited depth of the tree to find the optimal split gain node, which ensures efficiency and prevents overfitting [39].

Of the selected important features, most were GLCM texture features and shape features, which could reflect the heterogeneity within the ROI. In the three-classification task, the SWI_GLCM correlation of SN was consistently selected in each fold, indicating its significance in differentiating PD from MSA and HCs. It is generally accepted that the degeneration and depiction of neurons in the SN is the most important reason behind PD symptoms [40]. Based on that, the ‘swallow tail’ sign on MRI is considered to be valuable in identifying PD patients [41]. Iron deposition in SN causes heterogeneity of SN on SWI. As a consequence, SWI_GLCM correlation of SN plays a central role in distinguishing between PD, MSA patients and HCs. Except for the three-classification task, the shape_flatness of GP emerges as the most valuable feature in all the other tasks, regardless of single or combination sequence. The shape features represent the volume, area, or shape of the GP. Han et al. explored different iron deposition patterns between MSA and PSP, and found that MSA-P patients had iron deposition in posterolateral PUT and adjacent lateral aspect of GP [42]. Besides, researchers found that both segments of GP suffered from dopamine loss in Parkinsonism symptoms [43]. Furthermore, Pereira et al. noted that the firing rates of neurons in the GP, specifically the internal segment of the GP internus, exhibit differences between patients with MSA and PD [44]. GP is a central hub in nigro-pallidal dopamine pathway. Different segment of GP, which contains internal of GP (GPi) and external of GP (GPe), plays a different role in nigro-pallidal projection and is related to various motor and non-motor symptoms in PD patients [45]. It has been suggested that the brains of MSA patients may experience more severe shape alteration, such as atrophy and shrinkage, than PD patients, particularly in the later stages. However, this conclusion requires further confirmation through large-scale and multi-center studies. In addition, there is increasing evidence that the iron-specific deposit pattern of PUT is more prevalent in MSA patients, especially in MSA-P patients [15, 29]. On the basis of it, putaminal hypointensity on SWI has been proposed as a specific sign for MSA-p patients [4]. The inclusion of both MSA-P and MSA-C (cerebellar variants of MSA) groups in our study could account for the discrepancy observed, as it may have reduced the specificity of differences in the PUT region. Additionally, a study has demonstrated that hypointensity in the PUT can also be observed in the elderly, so further studies with larger cohorts are necessary to assess the value of putaminal hypointensity in effectively differentiating MSA patients [46].

Our study has several limitations. Firstly, diagnoses of PD and MSA patients were made by experienced clinicians without pathology-confirmed evidence, but the consensus operational clinical diagnostic criteria and more than 2 years of the average follow-up period were used to guarantee the accurate diagnosis. Secondly, we did not classify MSA into different subtypes (MSA-P and MSA-C) for that MSA sample size was relatively small (*n* = 73), further studies will be conducted to analyze the MSA subtypes when more patients are recruited. Thirdly, although two neuroradiologists delineated independently, manual segmentation on SWI may introduce personal bias, particularly on the borders of basal nuclei. We used the ICCs to make sure that the segmentation of ROIs in our study has a good reliability, all VOIs with ICCs ≥0.8 in our study.

Our study findings demonstrated that radiomics analysis, utilizing a combination of conventional sequences such as T1WI and SWI, holds promise in differentiating PD from MSA. This finding provides strong support for the accurate diagnosis at an early stage, and further research should focus on developing straightforward and efficient methods for disease classification.

### Supplementary Information

**Additional file 1.**

## Data Availability

The data that support the findings of this study are available from the corresponding author upon reasonable request.
