# Supplementary material for: Multi-parametric radiomics of conventional T1 weighted and susceptibility-weighted imaging for differential diagnosis of idiopathic Parkinson’s disease and multiple system atrophy
Source: BMC Med Imaging. 2023 Dec 8;23:204. doi: 10.1186/s12880-023-01169-1 (PMC10709839; doi:10.1186/s12880-023-01169-1)
Supplement: Supplementary file 1 — Additional file 1. [file 12880_2023_1169_MOESM1_ESM.docx]

**Supplementary Table1**. The performance of different models using T1 and SWI sequence in three-classification tasks (PD vs MSA vs HC) across five folds

| sequence | | models | \| Mean±SD \| \| \| \| \| --- \| --- \| --- \| --- \| \| Sen \| Spec \| ACC \| AUC \| \| |
| --- | --- | --- | --- | --- | --- | --- | --- | --- | --- | --- | --- | --- |
| SWI | \| LR \| \| --- \| \| SVM \| \| LGBM \| | | \| 0.744±0.015 \| 0.868±0.012 \| 0.751±0.012 \| 0.845±0.016 \| \| \| --- \| --- \| --- \| --- \| --- \| \| 0.765±0.025 \| 0.874±0.016 \| 0.757±0.020 \| \| 0.885±0.019 \| \| \| 0.770±0.017 \| 0.878±0.016 \| 0.778±0.012 \| 0.894±0.016 \| \| \| |
| T1 | \| LR \| \| --- \| \| SVM \| \| LGBM \| | | \| 0.716±0.016 \| 0.861±0.009 \| 0.729±0.010 \| 0.814±0.015 \| \| --- \| --- \| --- \| --- \| \| 0.677±0.023 \| 0.851±0.011 \| 0.712±0.008 \| 0.850±0.021 \| \| 0.694±0.024 \| 0.852±0.011 \| 0.721±0.007 \| 0.855±0.020 \| |
| SWI+T1 | \| LR \| \| --- \| \| SVM \| \| LGBM \| | | \| 0.729±0.013 \| 0.862±0.013 \| 0.737±0.008 \| 0.867±0.015 \| \| --- \| --- \| --- \| --- \| \| 0.763±0.030 \| 0.890±0.009 \| 0.791±0.010 \| 0.882±0.027 \| \| 0.800±0.009 \| 0.870±0.009 \| 0.801±0.012 \| 0.890±0.011 \| |

Note: Sen, sensitive; Spec, specificity; ACC, accuracy; AUC, area under the curve; SWI, susceptibility weighted imaging; T1, T1 weighted imaging; LR, logistic regression; SVM, support vector machine; LGBM, light gradient boosting machine.

**Supplementary Table2.** The performance of different models using T1 and SWI sequence in binary classification tasks (PD vs MSA) across five folds

| sequence | | models | \| Mean±SD \| \| \| \| \| --- \| --- \| --- \| --- \| \| Sen \| Spec \| ACC \| AUC \| \| |
| --- | --- | --- | --- | --- | --- | --- | --- | --- | --- | --- | --- | --- |
| SWI | \| LR \| \| --- \| \| SVM \| \| LGBM \| | | \| 0.734±0.016 \| 0.827±0.016 \| 0.774±0.011 \| 0.846±0.014 \| \| \| --- \| --- \| --- \| --- \| --- \| \| 0.696±0.015 \| 0.790±0.020 \| 0.747±0.016 \| \| 0.822±0.014 \| \| \| 0.752±0.013 \| 0.869±0.016 \| 0.807±0.006 \| 0.865±0.018 \| \| \| |
| T1 | \| LR \| \| --- \| \| SVM \| \| LGBM \| | | \| 0.715±0.016 \| 0.760±0.015 \| 0.728±0.008 \| 0.805±0.016 \| \| --- \| --- \| --- \| --- \| \| 0.656±0.021 \| 0.864±0.025 \| 0.747±0.014 \| 0.836±0.014 \| \| 0.692±0.021 \| 0.833±0.021 \| 0.749±0.013 \| 0.833±0.007 \| |
| SWI+T1 | \| LR \| \| --- \| \| SVM \| \| LGBM \| | | \| 0.806±0.014 \| 0.772±0.013 \| 0.771±0.011 \| 0.831±0.011 \| \| --- \| --- \| --- \| --- \| \| 0.741±0.025 \| 0.808±0.010 \| 0.782±0.011 \| 0.854±0.015 \| \| 0.836±0.015 \| 0.872±0.016 \| 0.831±0.014 \| 0.871±0.009 \| |

Note: Sen, sensitive; Spec, specificity; ACC, accuracy; AUC, area under the curve; SWI, susceptibility weighted imaging; T1, T1 weighted imaging; LR, logistic regression; SVM, support vector machine; LGBM, light gradient boosting machine.
